# Causal Relationship Between Gut Microbiome and Infectious Mononucleosis: Bidirectional Mendelian Randomization Reveals Infectious Mononucleosis-Driven Gut Dysbiosis

**DOI:** 10.7759/cureus.111983

**Published:** 2026-07-03

**Authors:** Yuanxiao Li, Jiayan Wang, Ni Zhang, Xiaonan Xu, Xingxing Dai, Yan Li, Li Cheng, Hui Liu, Pengcheng Ren, Hanwei Ma

**Affiliations:** 1 Department of Pediatric Gastroenterology, The Second Hospital of Lanzhou University, Lanzhou, CHN

**Keywords:** ebv infection, gut microbiome, infectious mononucleosis, mendelian randomization, pediatrics

## Abstract

Background

To assess bidirectionality between gut microbiota and Epstein-Barr virus (EBV)-driven infectious mononucleosis (IM), we conducted two-sample Mendelian randomization (MR). Given IM’s heterogeneous symptoms and evidence linking microbiota to viral infection, this may inform novel prevention or treatment strategies.

Methods

We employed a bidirectional two-sample MR framework using summary data from 207 gut microbial taxa and 205 metabolic pathways (Dutch Microbiome Project, n = 7,738) and the FinnGen consortium. Causality was assessed via inverse variance weighting (IVW), MR-Egger, weighted median, and mode-based estimation. Statistical significance was set at P <0.05 (Bonferroni-corrected), with instruments validated by F-statistics >10. Heterogeneity and pleiotropy were evaluated using Cochran’s Q, MR-Egger intercepts, and leave-one-out analyses.

Results

Forward MR: Several bacterial pathways and taxa were positively associated with IM risk. These include the de novo purine nucleotide biosynthesis II superpathway (odds ratio (OR) = 1.246, 95% confidence interval (CI): 1.026-1.514, P = 0.027), anhydromuropeptide recycling (OR = 1.24, 95% CI: 1.014-1.517, P = 0.036), the superpathway of unsaturated fatty acid biosynthesis (*Escherichia coli*) (OR = 1.194, 95% CI: 1.019-1.4, P = 0.028), *Lactobacillaceae* (OR = 1.109, 95% CI: 1.02-1.206, P = 0.016), and *Lactobacillus* (OR = 1.108, 95% CI: 1.017-1.207, P = 0.019). Conversely, several taxa and pathways exhibited protective effects. These include the glucose-1-phosphate degradation pathway (G1P-DP) (OR = 0.852, 95% CI: 0.731-0.994, P = 0.042), heme biosynthesis from glutamate (OR = 0.809, 95% CI: 0.676-0.969, P = 0.032), the superpathway of L-tyrosine biosynthesis (OR = 0.9, 95% CI: 0.811-0.998, P = 0.046), the flavin biosynthesis I pathway (OR = 0.817, 95% CI: 0.672-0.993, P = 0.042),* Streptococcaceae* (OR = 0.869, 95% CI: 0.779-0.968, P = 0.011), and* Streptococcus* (OR = 0.844, 95% CI: 0.731-0.937, P = 0.020).

Reverse MR: IM was found to causally alter gut microbiome composition. IM was associated with a decrease of beneficial genera such as *Roseburia* (OR = 0.901, 95% CI: 0.837-0.982, P = 0.016) and *Bacteroides ovatus* (OR = 0.915, 95% CI: 0.841-0.995, P = 0.038), as well as *Streptococcus* (OR = 0.876, 95% CI: 0.775-0.991, P = 0.035). Conversely, IM increased the risk of enrichment for *Prevotellaceae* (OR = 1.107, 95% CI: 1.016-1.210, P = 0.020) and *Prevotella copri* (OR = 1.096, 95% CI: 1.000-1.200, P = 0.048). Regarding metabolic pathways, IM increased the risk of polyamine biosynthesis II (OR = 1.123, 95% CI: 1.016-1.243, P = 0.024), L-lysine biosynthesis II (OR = 1.094, 95% CI: 1.008-1.118, P = 0.031), and L-lysine biosynthesis VI (OR = 1.083, 95% CI: 1.000-1.172, P = 0.048), while showing a protective association with L-rhamnose degradation I (OR = 0.922, 95% CI: 0.851-0.999, P = 0.046).

Conclusion

This study provides genetic evidence of a bidirectional causal relationship between the gut microbiome and IM. These findings suggest that IM may influence gut microbial ecosystem structure, characterized by a reduction in beneficial symbionts (e.g., *Roseburia*) and an enrichment of potentially pro-inflammatory taxa (e.g., *P. copri*). These findings may inform future microbiota-targeted interventions or risk stratification strategies for EBV-related diseases. Limitations include the European ancestry of study populations and the need for mechanistic validation.

## Introduction

Acting as the human "second genome," the gut microbiome critically influences host metabolism, immune function, and disease pathogenesis. Recent innovations in high-throughput sequencing and genome-wide association studies (GWAS) methodologies make it feasible to comprehensively explore the extent to which host genetic profiles shape the structure and biological functions of intestinal microorganisms. The Dutch Microbiome Project performed deep metagenomic sequencing on fecal samples from 7,738 participants, precisely annotating 207 microbial taxa (spanning family, genus, and species levels) and 205 bacterial metabolic pathways. The study demonstrated that host genetic variants significantly affect gut microbiome composition and function, and the resulting large-scale dataset has been publicly released, becoming a foundational resource for exploring causal relationships between the gut microbiome and complex diseases worldwide [1]. Additionally, interindividual differences in the immunogenicity of microbiota-derived lipopolysaccharides (LPS) may influence the risk of autoimmune diseases, highlighting the tight connection between the gut microbiome, host immunity, and metabolic function [2].

Mendelian randomization (MR) uses genetic variants as instrumental variables (IVs) to overcome confounding and reverse causation common in observational studies, with sensitivity analyses (MR-Egger, weighted median, leave-one-out) controlling for horizontal pleiotropy [3,4].MR uses genetic variants as IVs to overcome confounding and reverse causation common in observational studies, with sensitivity analyses (MR-Egger, weighted median, leave-one-out) controlling for horizontal pleiotropy [3,4].

Though this condition carries a benign prognosis for most affected people, some cases can progress to chronic active Epstein-Barr virus (EBV) infection and raise susceptibility to subsequent autoimmune illnesses, including multiple sclerosis [5,6]. Notably, clinical manifestations following EBV infection vary considerably among individuals, with only a fraction developing classical infectious mononucleosis (IM), suggesting that host factors, including immune status and the microecological environment, may modulate disease susceptibility and severity [6]. Current management of IM is primarily supportive, and no specific antiviral therapy is routinely recommended, highlighting the need for novel preventive or therapeutic approaches.

Increasing evidence indicates that the gut microbiota can influence viral infection outcomes by regulating local and systemic immune responses. For instance, germ-free or antibiotic-treated mice exhibit more severe influenza virus infections, whereas topical application of microbiota can enhance antiviral immunity through induction of interferon-λ [7,8]. However, from a genetic epidemiological perspective, the causal relationship between gut microbiome features and IM remains largely unexplored.

Therefore, leveraging GWAS summary data from 207 microbial taxa and 205 metabolic pathways, this study employed two-sample MR to systematically probe the causal associations between gut microbiome traits and IM risk. By selecting valid genetic instruments and performing multiple sensitivity analyses, our present work seeks to reduce biases induced by horizontal pleiotropy, furnish genetic proof to validate the causal contribution of intestinal microbes to EBV-linked illnesses, and supply theoretical references for microbiota-centered intervention regimens for IM.

## Materials and methods

Ethical approval

This MR study utilized publicly available, de-identified summary-level genetic data. The original GWAS that generated these data obtained informed consent from all participants and received ethical approval from their respective institutional review boards. Since the present study involved only the secondary analysis of these aggregated, anonymized data, it did not require separate ethical approval. All data sources and their associated ethical approvals are explicitly cited within the manuscript.

Data sources for gut microbiome and IM

We extracted aggregated GWAS results relevant to gut microbial profiles from the Dutch Microbiome Project cohort, which analyzed 207 microbial taxa and 205 metabolic pathways to characterize microbiome composition and function. This population-based cohort included 7,738 participants from the northern Netherlands, all of whom had quality-controlled genotype and microbiome data, alongside body mass index (BMI) measurements. The study population was 58.1% female gender, with a mean age of 48.5 years (range: 8-84) and a mean BMI of 25.58 kg/m² (range: 13.10-63.70). For IM, GWAS data were acquired from the FinnGen consortium, a large-scale public-private partnership aiming to generate medical insights by analyzing genomic and health records from 500,000 Finnish biobank donors. Comprehensive details regarding the GWAS datasets utilized in this MR study are provided in Appendix.

The assumptions of an MR study

**Figure 1:**
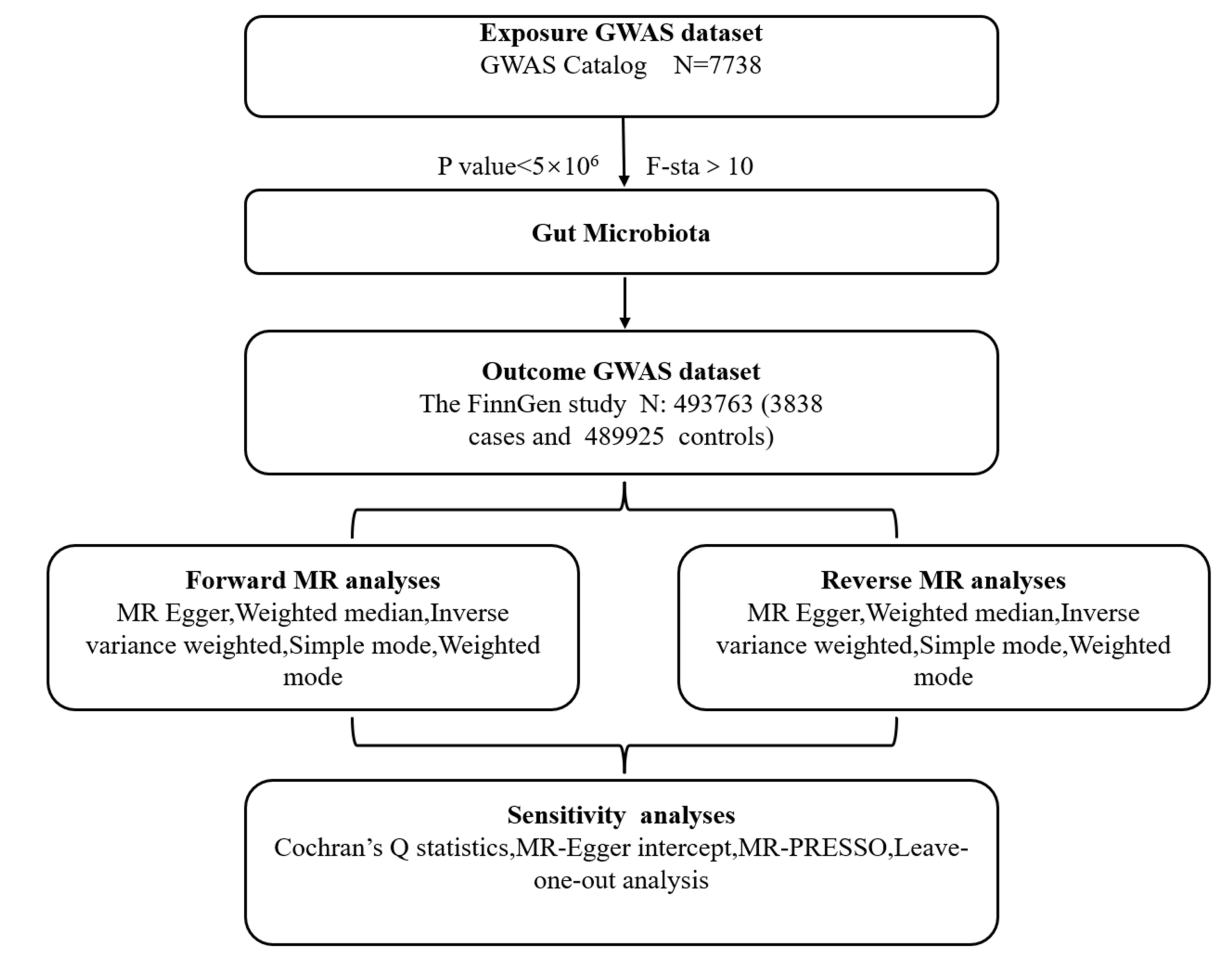
Flowchart of this study GWAS, genome-wide association studies; MR, Mendelian randomization.

A valid MR study is predicated on three core assumptions, as schematically depicted in Figure 1.

First, IVs must demonstrate a strong correlation with the target exposure. Instrument strength is typically evaluated via the F-statistic, defined as $F = \frac{R^2(n-k-1)}{k(1-R^2)}$, where R² denotes the variance explained by the IVs, n is the sample size, and k represents the number of IVs [9]. To prevent weak instrument bias, we applied a stringent threshold of F >10 to guarantee statistical reliability. Second, the chosen IVs must be independent of any unmeasured confounding factors affecting the exposure-outcome relationship. Third, IVs should influence the outcome exclusively through the exposure, thereby excluding horizontal pleiotropy. This ensures that genetic variants affect the outcome only via the intended causal pathway, avoiding biases from alternative routes. Summary statistics for the IVs associated with gut microbiota and IM are detailed in Appendix.

MR analysis

Causal associations among 207 taxa, 205 metabolic pathways, and IM were evaluated using multiple MR approaches. Statistical significance was defined as a two-sided P < 1.21 × 10⁻⁴ after Bonferroni correction (0.05/412) for gut microbiota analyses; P-values between 0.05 and this threshold were regarded as suggestive evidence of association. Reverse MR was implemented to confirm causal direction, applying the same analytical framework as in forward MR, with P <0.05 considered significant. Four complementary MR models -- MR-Egger, weighted median, random-effects inverse variance weighting (IVW), and weighted mode -- were employed. MR-Egger provides unbiased causal estimates even under widespread pleiotropy, assuming genetic variant-exposure associations are independent of such effects. The weighted median approach requires that ≥50% of analytical weight arises from valid IVs. IVW functions as a meta-analytic method assuming exclusive mediation via the exposure, whereas the weighted mode remains robust when the largest cluster of instruments yields consistent causal estimates, even if most others are invalid. A suggestive causal link was inferred when any IVW-based analysis yielded P <0.05. Significant findings underwent sensitivity analyses to evaluate heterogeneity and pleiotropy. Cochran’s Q test assessed heterogeneity, and the MR-Egger intercept addressed horizontal pleiotropy (P>0.05 implying absence). Leave-one-out analysis was also conducted, iteratively excluding individual single-nucleotide polymorphisms (SNPs) and recalculating IVW estimates to ensure no single variant disproportionately influenced results.

All computations were executed in R (version 4.5.0; R Foundation for Statistical Computing, Vienna, Austria) with the TwoSampleMR (version 0.6.17; MRC Integrative Epidemiology Unit, University of Bristol, Bristol, UK) and stats (version 4.5.0; R Foundation for Statistical Computing, Vienna, Austria) packages.

## Results

Causal relationship between gut microbiome and IM

The causal architecture connecting the gut microbiome to IM was characterized using IVW MR. Evidence from the discovery set pointed to specific microbial pathways and taxa conferring significant risk or protection against IM. Comprehensive sensitivity tests, alongside the main statistical outputs (Appendix), substantiated the reliability of these observed causal relationships.

**Figure 2:**
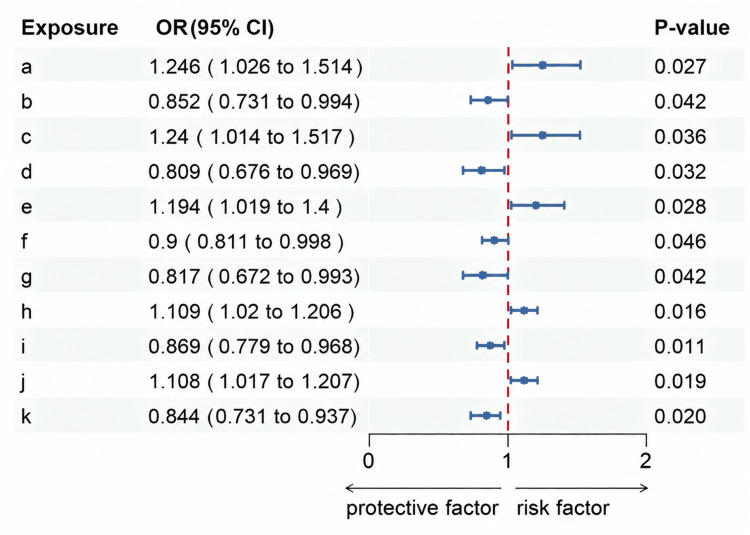
Gut microbiome associated with IM a: The de novo purine nucleotide biosynthesis II superpathway. b: The glucose-1-phosphate degradation pathway (G1P-DP). c  Anhydromuropeptide recycling. d: Heme biosynthesis from glutamate. e: Superpathway of unsaturated fatty acid biosynthesis (*Escherichia coli*). f: Superpathway of L-tyrosine biosynthesis. g: RBOSYN2 - PWY (flavin biosynthesis I pathway) in bacteria and plants. h: Family *Lactobacillaceae*. i: Family *Streptococcaceae*. j: Genus *Lactobacillus*. k: Genus *Streptococcus*. IM, infectious mononucleosis.

Using MR analysis, we investigated the causal associations between gut microbiome features and IM. Multiple metabolic pathways and microbial taxa were identified as being significantly associated with the risk of IM (presented in the forest plot). Specifically, the de novo purine nucleotide biosynthesis II superpathway (odds ratio (OR) = 1.246, 95% confidence interval (CI): 1.026-1.514, P = 0.027), anhydromuropeptide recycling (OR = 1.24, 95% CI: 1.014-1.517, P = 0.036), superpathway of unsaturated fatty acid biosynthesis (*Escherichia coli*) (OR = 1.194, 95% CI: 1.019-1.4, P = 0.028), family *Lactobacillaceae* (OR = 1.109, 95% CI: 1.02-1.206, P = 0.016), and genus *Lactobacillus* (OR = 1.108, 95% CI: 1.017-1.207, P = 0.019) were positively associated with an increased risk of IM (OR > 1, 95% CI excluding 1). In contrast, the glucose‑1‑phosphate degradation pathway (G1P‑DP) (OR = 0.852, 95% CI: 0.731-0.994, P = 0.042), heme biosynthesis from glutamate (OR = 0.809, 95% CI: 0.676-0.969, P = 0.032), superpathway of L‑tyrosine biosynthesis (OR = 0.9, 95% CI: 0.811-0.998, P = 0.046), RBOSYN2‑PWY (flavin biosynthesis I pathway) in bacteria and plants (OR = 0.817, 95% CI: 0.672-0.993, P = 0.042), family *Streptococcaceae* (OR = 0.869, 95% CI: 0.779-0.968, P = 0.011), and genus *Streptococcus* (OR = 0.844, 95% CI: 0.731-0.937, P = 0.020) were negatively associated with the disease risk (OR < 1, 95% CI excluding 1), indicating their protective effects against IM. Sensitivity analyses for the causal association between the gut microbiome and IM are presented in Appendix. The forward forest plot is shown in Figure 2.

**Figure 3:**
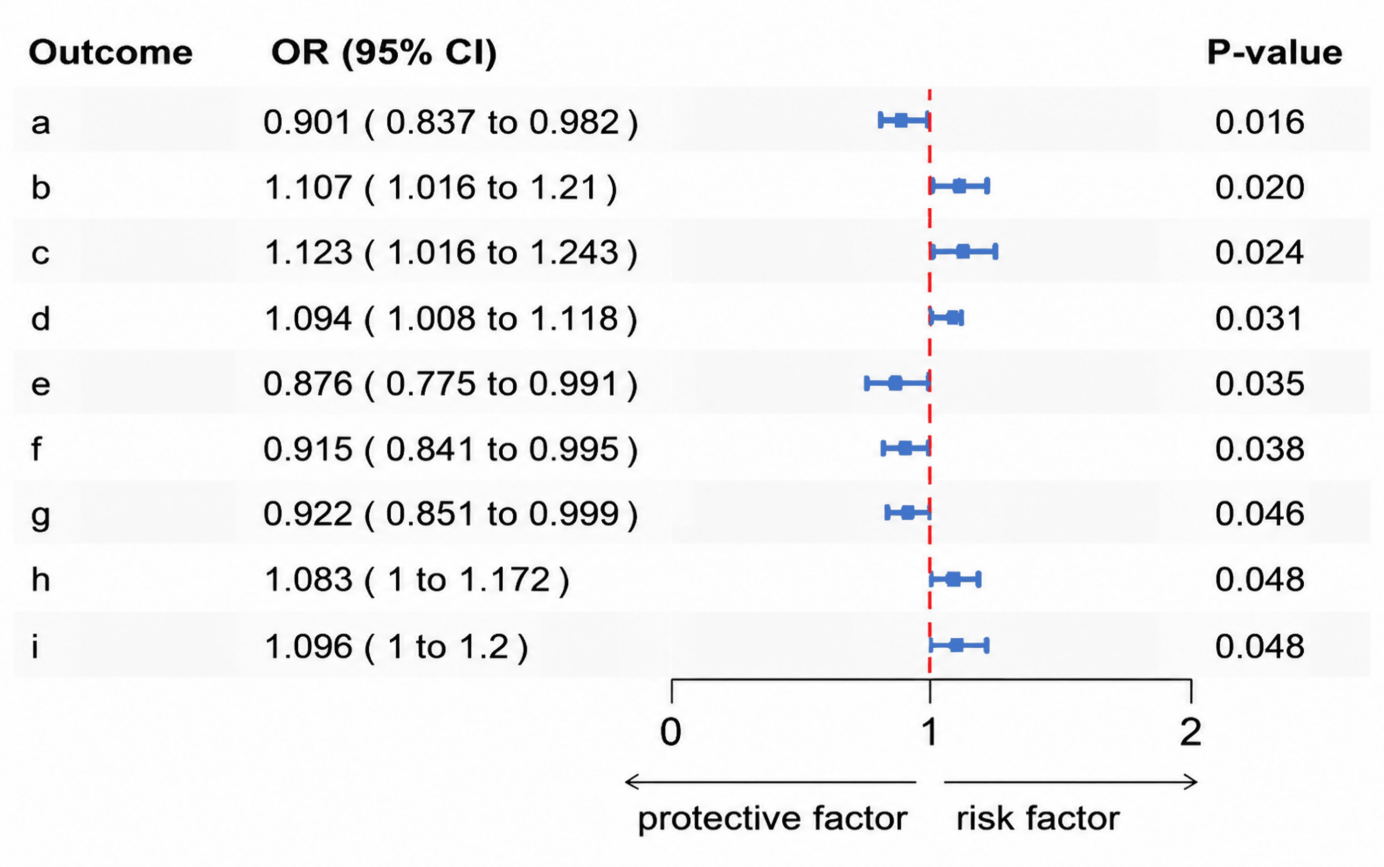
IM associated with the gut microbiome a: Genus *Roseburia*. b: Family *Prevotellaceae*. c: Superpathway of polyamine biosynthesis II. d: L-lysine biosynthesis II. e: Genus *Streptococcus*. f:​ Species *Bacteroides ovatus. *g: L-rhamnose degradation I. h: L-lysine biosynthesis VI. I: Species *Prevotella copri. * IM, infectious mononucleosis.

MR analysis was performed to assess the causal effects of IM on gut microbial taxa and metabolic pathways. The MR results for the causal effect of childhood allergy on the gut microbiome in the discovery dataset are presented in Appendix. The odds ratios (ORs) with 95% confidence intervals (CIs) and the corresponding P-values are presented as follows. For gut microbial genera, IM exhibited a significant protective effect on Genus *Roseburia* (OR = 0.901, 95% CI: 0.837-0.982, P = 0.016) and Genus *Streptococcus* (OR = 0.876, 95% CI: 0.775-0.991, P = 0.035). In contrast, it served as a significant risk factor for Family *Prevotellaceae* (OR = 1.107, 95% CI: 1.016-1.210, P = 0.020) and Species *Prevotella copri* (OR = 1.096, 95% CI: 1.000-1.200, P = 0.048). Additionally, IM showed a protective association with Species *Bacteroides ovatus* (OR = 0.915, 95% CI: 0.841-0.995, P = 0.038). Regarding gut microbial metabolic pathways, IM significantly increased the risk of the superpathway of polyamine biosynthesis II (OR = 1.123, 95% CI: 1.016-1.243, P = 0.024), L‑lysine biosynthesis II (OR = 1.094, 95% CI: 1.008-1.118, P = 0.031), and L‑lysine biosynthesis VI (OR = 1.083, 95% CI: 1.000-1.172, P = 0.048). Meanwhile, it exerted a protective effect against L‑rhamnose degradation I (OR = 0.922, 95% CI: 0.851-0.999, P = 0.046). IVs exhibited homogeneity (Cochran Q: P > 0.05). Sensitivity analyses for the causal association between the IM and gut microbiome are presented in Appendix. No evidence of horizontal pleiotropy was detected (MR-Egger intercept and MR-PRESSO: P > 0.05). The reverse forest plot is shown in Figure 3.

## Discussion

No previous MR work has extensively assessed causal links between IM and gut microbiome taxonomy alongside metabolic signaling pathways to the same degree, making our analysis one of the earliest relevant explorations. Our findings indicate that IM may remodel the gut microbiota ecosystem, marked by a depletion of beneficial commensals, an enrichment of potentially pro-inflammatory taxa, and alterations in microbial metabolic functions. These results offer novel insights into the putative interplay between EBV-mediated immunity and the gut microbiome.

Among the microbial taxa identified, IM was associated with a decreased abundance of the genus *Roseburia*. This genus is a well-recognized butyrate producer and is widely regarded as a key indicator of intestinal health [10]. Numerous earlier investigations verify that *Roseburia* strains largely drive butyrate synthesis within the colon. As an indispensable fuel for colonocytes, this short-chain fatty acid exerts core functions in sustaining intact gut epithelial barriers and stabilizing intestinal immune status [11,12]. Beyond that, prior experiments have proven that butyrate can boost the differentiation process of regulatory T cells [13]. A reduction in *Roseburia* abundance has been consistently documented in inflammatory bowel disease, metabolic syndrome, and other chronic inflammatory conditions [14,15]. Therefore, the observed decline plausibly reflects that IM-related immune activation fosters a less favorable intestinal microenvironment.

*Streptococcus* belongs to the family *Streptococcaceae* as a subordinate taxonomic unit. Forward MR analyses suggest that taxa of *Streptococcus* and *Streptococcaceae* are causally associated with a reduced risk of IM, whereas reverse MR indicates that IM can decrease the relative abundance of *Streptococcus*. While specific *Streptococcus* species are established opportunistic pathogens, a substantial proportion of commensal *streptococci* play crucial roles in mediating colonization resistance and preserving microbial homeostasis [16]. Notably, acute EBV infection triggers profound T-cell activation and cytokine production [17], which may consequently reshape the gastrointestinal microbial niche. However, the precise biological implications of these alterations remain to be fully elucidated and warrant further investigation.

Conversely, IM was characterized by an increased abundance of Family *Prevotellaceae*. Accumulating evidence suggests that *P. copri* may function as a pathobiont under specific host conditions [18]. Scher et al. reported that intestinal expansion of *P. copri* correlates with enhanced susceptibility to rheumatoid arthritis [19]. Subsequent studies have demonstrated that *P. copri* can elicit antigen-specific immune responses and promote pro-inflammatory pathways, particularly those mediated by Th1 and Th17 cells [20,21]. It is worth highlighting that EBV infection contributes to the pathological progression of a range of autoimmune illnesses, exemplified by rheumatoid arthritis, multiple sclerosis, and systemic lupus erythematosus [22]. Thus, the enrichment of *Prevotella*-related taxa observed in our study may represent a potential mechanism linking EBV-associated immune activation to long-term immune dysregulation.

This study demonstrated that IM can reduce the abundance of *B. ovatus*. As a prevalent commensal species, *B. ovatus* plays a pivotal role in polysaccharide degradation and short-chain fatty acid production [23]. Evidence suggests that it enhances intestinal immune homeostasis by stimulating IL-22 production [24]. Given its capacity to bolster mucosal barrier integrity and beneficial host-microbe crosstalk, *B. ovatus* is considered a promising candidate for next-generation probiotics [25]. Consequently, the observed decline in this species likely signifies an impairment of protective microbial functions during IM.

IM is associated with an increased abundance of *P. copri*. IM may indirectly induce alterations in gut microbiota composition (dysbiosis), potentially mediated by systemic immune dysregulation and inflammatory responses. Within this context of dysbiosis, specific lineages of *P. copri* may undergo overgrowth or exhibit altered metabolite profiles, thereby further exacerbating or modulating the host's immune response [26].

The genus *Lactobacillus* is a member of the family *Lactobacillaceae* and serves as its type genus. Review articles have indicated that lactobacilli exert protective effects against a range of diseases, including viral infections, by mitigating inflammation and modulating immune responses. Thus, *lactobacilli *may reduce the incidence of IM [27].

We identified significant alterations in several microbial metabolic pathways in patients with IM. Notably, a comprehensive literature review reveals no prior evidence directly linking these specific pathways to IM clinical outcomes. We therefore propose that the observed dysregulation of these pathways does not reflect primary disease mechanisms but rather represents secondary perturbations of the gut microbiota during acute IM. Acute EBV infection is frequently accompanied by systemic inflammation, transient immunosuppression, altered dietary intake due to pharyngitis and fatigue, and occasional empirical antibiotic use, all of which are established drivers of gut microbial community restructuring. The concurrent enrichment of bacterial housekeeping pathways involved in nucleotide, amino acid, cofactor, and cell wall metabolism likely captures a broad shift in microbial community composition and metabolic activity, such as the expansion of facultative anaerobes within the *Enterobacteriaceae* family, rather than pathway-specific contributions to viral replication or immune dysregulation [28].

Additionally, we acknowledge the potential influence of database annotation bias. Functional profiling tools applied to human-associated metagenomic data often assign conserved protein domains to well-characterized microbial reference pathways, which may overrepresent bacterial metabolism even when signals originate from low-abundance taxa or residual environmental DNA. This is particularly relevant for highly specialized pathways such as anhydromuropeptide recycling and *E. coli*-specific fatty acid biosynthesis, whose detection in non-stool samples should be interpreted with caution and validated against negative controls.

Despite the utilization of an MR framework to mitigate confounding and reverse causation, several limitations warrant consideration. First, our heavy utilization of GWAS statistics from European-descent participants hinders the broad applicability of our research outcomes across different ethnic populations. Second, while sensitivity tests failed to detect prominent horizontal pleiotropy, leftover pleiotropic influences are not completely avoidable. Third, the intrinsic biological pathways we identified, especially lysine biosynthesis and rhamnose degradation, lack comprehensive mechanistic validation, so corresponding conclusions should be interpreted prudently. Fourth, as our conclusions are derived from genetic inference, validation in prospective cohorts and experimental models is essential to establish causality and elucidate molecular mechanisms. Fifth, potential database annotation bias may lead to overrepresentation of well-characterized bacterial pathways; thus, detection of specialized pathways in non-stool samples should be interpreted with caution and validated against negative controls. Finally, distinguishing host-driven metabolic reprogramming from microbially derived signals remains challenging, and observed dysbiosis may reflect secondary perturbations driven by systemic inflammation, dietary changes, or antibiotic use during acute infection rather than primary pathogenic mechanisms.

## Conclusions

In summary, this MR analysis provides genetic evidence suggesting that IM may causally influence gut microbial composition and metabolic functions. Specifically, IM was associated with decreased abundance of potentially beneficial taxa (e.g., *Roseburia* and *B. ovatus*), increased abundance of *Prevotellaceae* and *P. copri*, and alterations in microbial pathways related to polyamine and amino acid metabolism. These findings offer novel insights into the complex interplay between EBV-associated disease and the gut microbiome, highlighting the potential for microbiota-targeted therapeutic strategies. Nevertheless, given the aforementioned limitations, future studies integrating paired host transcriptomics, strain-resolved metagenomics, and targeted metabolite quantification in prospective cohorts are warranted to determine whether these microbial shifts exert modulatory effects on host immunity or merely represent epiphenomena of acute infection.

## Appendices

Tables and figures pertaining to the Mendelian randomization analyses are provided in the Appendix.

The dataset is available on Microsoft OneDrive​ at: https://1drv.ms/f/c/bb68d38b3c962f91/IgAep4WYhcSmR5juSfMFJ3-uAeNYZDKCCrg-2-8vZR98MtI?e=MQrVZl.
